# Quantification of Viable *Brochothrix thermosphacta* in Cold-Smoked Salmon Using PMA/PMAxx-qPCR

**DOI:** 10.3389/fmicb.2021.654178

**Published:** 2021-07-14

**Authors:** Agnès Bouju-Albert, Sabrina Saltaji, Xavier Dousset, Hervé Prévost, Emmanuel Jaffrès

**Affiliations:** UMR 1014, Secalim, INRAE, Oniris, Nantes, France

**Keywords:** viable, *Brochothrix thermosphacta*, spoilage, smoked salmon, PMA, PMAxx-based qPCR, rpoC gene

## Abstract

The aim of this study was to develop a rapid and accurate PMA-qPCR method to quantify viable *Brochothrix thermosphacta* in cold-smoked salmon. *B. thermosphacta* is one of the main food spoilage bacteria. Among seafood products, cold-smoked salmon is particularly impacted by *B. thermosphacta* spoilage. Specific and sensitive tools that detect and quantify this bacterium in food products are very useful. The culture method commonly used to quantify *B. thermosphacta* is time-consuming and can underestimate cells in a viable but not immediately culturable state. We designed a new PCR primer set from the single-copy *rpoC* gene. QPCR efficiency and specificity were compared with two other published primer sets targeting the *rpoC* and *rpoB* genes. The viability dyes PMA or PMAxx were combined with qPCR and compared with these primer sets on viable and dead *B. thermosphacta* cells in BHI broth and smoked salmon tissue homogenate (SSTH). The three primer sets displayed similar specificity and efficiency. The efficiency of new designed *rpoC* qPCR on viable *B. thermosphacta* cells in SSTH was 103.50%, with a linear determination coefficient (r^2^) of 0.998 and a limit of detection of 4.04 log CFU/g. Using the three primer sets on viable cells, no significant difference was observed between cells treated or untreated with PMA or PMAxx. When dead cells were used, both viability dyes suppressed DNA amplification. Nevertheless, our results did not highlight any difference between PMAxx and PMA in their efficiency to discriminate viable from unviable *B. thermosphacta* cells in cold-smoked salmon. Thus, this study presents a rapid, specific and efficient *rpoC*-PMA-qPCR method validated in cold-smoked salmon to quantify viable *B. thermosphacta* in foods.

## Introduction

*Brochothrix thermosphacta* is one of the main food spoilage bacteria, and can cause important economic losses in the food industry. This bacterium can produce off-odors leading to food waste, which moreover contributes to the ecological impact of food spoilage (Remenant et al., 2015; Illikoud et al., 2018a). In beef for example, *B. thermosphacta* can produce dairy – cheesy and creamy – off-odors (Dainty and Mackey, 1992; Casaburi et al., 2014). Seafood products, whose consumption is constantly increasing worldwide, can also be spoiled by *B. thermosphacta*, such as cooked and peeled shrimp with the production of strong butter, buttermilk-like, sour, and nauseous off-odors (Mejlholm et al., 2005; Laursen et al., 2006; Jaffrès et al., 2011). In cold-smoked salmon, *B. thermosphacta* can also produce butter/plastic/rancid, blue-cheese, sour/pungent off-odors (Joffraud et al., 2001, 2006; Stohr et al., 2001). *B. thermosphacta* has been described as widely disseminated along the food chain, from the raw material to the final product and during storage until use by date. This bacterium has also been isolated from food processing plants (from floors, walls, machines, etc.) which constitute one of the main sources of food contamination during processing (Stackebrandt and Jones, 2006; Nychas et al., 2008).

In the field of microbiological analysis, the non-cultural molecular methods such as quantitative real-time PCR (qPCR) have become essential tools in the last decade to detect and quantify microorganisms, with high precision in complex microbiota, e.g., in food matrices or food processing plants (Kuchta et al., 2014; Franco-Duarte et al., 2019). Quantitative real-time PCR (qPCR) is specific and fast tool to detect and quantify microorganisms, in complex microbiota, e.g., in food matrices or food processing plants (Kuchta et al., 2014). The qPCR detects bacteria in a viable but not immediately culturable state (VBNC), which are underestimates when using culturable microbiological approaches (Postollec et al., 2011). Nevertheless, dead bacteria can be revealed and quantified by qPCR. The qPCR using viability dyes such as propidium monoazide (PMA-qPCR) has been developed. PMA used before DNA extraction can penetrate unviable/dead cells, bind to their DNA and subsequently inhibit the PCR amplification, ensuring a selective quantification of viable bacteria (Elizaquível et al., 2014). PMA-qPCR is largely used nowadays to efficiently detect and quantify viable bacteria in food, e.g., *Campylobacter* (Josefsen et al., 2010; Castro et al., 2018), *Listeria monocytogenes* (Pan and Breidt, 2007; Desneux et al., 2015), *Salmonella* (Liang et al., 2011; Fang et al., 2018), *Staphylococcus aureus* (Zhang et al., 2015; Dong et al., 2018), *Vibrio parahaemolyticus* (Zhu et al., 2012; Niu et al., 2018), *Escherichia coli* O157:H7 (Elizaquível et al., 2012; Zhou et al., 2017), *Photobacterium* (Macé et al., 2013), and *Brochothrix thermosphacta* (Pennacchia et al., 2009; Mamlouk et al., 2012). The choice of the targeted phylogenetic marker is determining when such molecular tools are used. The 16S ribosomal RNA gene, commonly used to identify bacterial species, presents biases because multiple copies of this gene are often present in bacterial genomes with possible sequence variations (e.g., genomes of *B. thermosphacta* strains can contain up to 9 copies of the 16S rRNA gene) (Paoli et al., 2017; Illikoud et al., 2018b). This can lead to mispriming affecting amplification efficiency or quantification reliability. The use of single-copy protein-coding genes such as *gyrB* (DNA gyrase subunit B) or *rpoB* and *rpoC* (RNA polymerase subunit B or C) for bacterial detection and quantification is an alternative to this limitation (Case et al., 2007; Macé et al., 2013; Fougy et al., 2016).

We designed a new *B. thermosphacta rpoC* gene primer set, and compared its PMA-qPCR efficiency with the efficiency of two previously published *rpoB* or *rpoC* gene primer sets. We compared the efficiency of PMA or PMAxx treatments and described a rapid assay to quantify viable *B. thermosphacta* using PMA-qPCR with the new *rpoC* primer set. The method was successfully used to quantify *B. thermosphacta* in artificially contaminated cold-smoked salmon. To our knowledge, this study is a first about the use of qPCR to quantify the spoilage bacterium *B. thermosphacta* in combination with the use of a viability dye (PMA or PMAxx) and the targeting of a single-copy gene (*rpoC* or *rpoB*).

## Materials and Methods

### Bacterial Strains and *B. thermosphacta* Enumeration

A total of 26 strains belonging to 12 different bacterial species frequently isolated from various food products, notably cold-smoked salmon (CSS) or from a processing plant were used (Table 1). All strains were recovered from −80°C freezers on their appropriate agar media: Brain Heart Infusion agar (BHI) (VWR Chemicals Prolabo, France); BHI + 2% NaCl (Merck, France); de Man, Rogosa and Sharpe medium (MRS) (Biokar Diagnostics, France); Elliker broth (Biokar Diagnostics, France) supplemented with 1.5% agar (Biokar Diagnostics, France). Each strain was sub-cultured from the colonies on the plates in its corresponding broth and incubated at its optimal temperature growth for 24 to 48 h (Table 1) to obtain bacterial suspensions. *B. thermosphacta* DSM 20171 in pure culture was enumerated on BHI agar after 48 h incubation at 25°C. *B. thermosphacta* inoculated in smoked salmon tissue homogenate (SSTH) were enumerated on streptomycin tallous acetate actidione (STAA) agar supplemented with the STAA selective supplement (Oxoid, France), and incubated at 25°C for 48 h. Total viable counts in SSTH samples were estimated on plate count agar (PCA) (Biokar, France) supplemented with 2% NaCl (Merck, France) and incubated at 25°C for 72 h.

**TABLE 1:** Bacterial strains used to assess the specificity of the qPCR primers.

Species	Strains	Isolated from	Incubation temperature (°C)	Growth medium^a^
*Brochothrix thermosphacta*	DSM 20171^T^	Fresh pork sausage	25	BHI
*Brochothrix thermosphacta*	Oniris 19/R/663	Smoked salmon	25	BHI
*Brochothrix thermosphacta*	CD 340 (a)	Shrimp	25	BHI
*Brochothrix thermosphacta*	EBP 3084 (b)	Salmon	25	BHI
*Brochothrix thermosphacta*	TAP 125 (c)	Chicken	25	BHI
*Brochothrix thermosphacta*	EBP 3017 (b)	Cod	25	BHI
*Brochothrix campestris*	DSM 4712^T^	Soil	25	BHI
*Carnobacterium maltaromaticum*	Oniris 19/R/671	Smoked salmon factory	30	Elliker
*Carnobacterium divergens*	NCDO 2763^T^	Vacuum-packaged beef	30	Elliker
*Enterococcus faecalis*	ATCC 19433^T^	Unknown	37	Elliker
*Enterococcus faecium*	CIP 54.33	Canned fish	37	Elliker
*Escherichia coli*	CIP 53.125	Human feces	37	BHI
*Companilactobacillus alimentarius*	DSM 20181	Marinated fish product	30	MRS
*Latilactobacillus sakei subsp. sakei*	DSM 20017^T^	Moto starter of sake	30	MRS
*Lactococcus piscium*	Oniris 19/R/684	Smoked salmon	25	Elliker
*Lactococcus raffinolactis*	Oniris MIP 2453	Unknown	25	Elliker
*Listeria monocytogenes*	DSM 12464 ^T^	Poultry	37	BHI
*Listeria monocytogenes*	CIP 78.35	Spinal fluid	37	BHI
*Listeria grayi*	CIP 68.18^T^	Feces of chinchilla	37	BHI
*Listeria innocua*	CIP 80.11^T^	Brain of cow	37	BHI
*Photobacterium phosphoreum*	CIP 102511^T^	Unknown	15	BHI 2% NaCl
*Pseudomonas fluorescens*	CIP 69.13^T^	Pre-filter tanks	30	BHI
*Psychrobacter sp.*	Oniris 19/R/675	Smoked salmon factory	25	BHI 2% NaCl
*Serratia liquefaciens*	ATCC 27592^T^	Milk	37	BHI
*Shewanella baltica*	Oniris 19/R/670	Smoked salmon factory	25	BHI 2% NaCl
*Staphylococcus epidermidis*	CIP 68.21	Unknown	37	BHI

*DSM, Deutsche Sammlung von Mikroorganismen and Zellkulturen GmbH, Braunschweig, Germany; CIP, Centre de Ressources Biologiques de l’Institut Pasteur, Paris, France; NCDO, National Collection of Dairy Organisms, Shinfield, United Kingdom; ATCC, American Type Culture Collection, Manassas (VA) United States; Oniris, Secalim Bacterial Culture Collection, Nantes, France. ^T^: Type Strain; BHI, Brain Heart Infusion agar (VWR Chemicals Prolabo, France); MRS, de Man Rogosa and Sharpe and Elliker media (Biokar Diagnostics, France). (a) Jaffrès et al. (2009), (b) Chaillou et al. (2015), (c) Illikoud et al. (2019).*

### Preparation of the Smoked Salmon Tissue Homogenate

Cold-smoked salmon was provided by a French company. *B. thermosphacta* was not detected in it using standard culture method on STAA, with the STAA selective supplement, and the real-time PCR methods developed in this study. The smoked salmon tissue homogenate (SSTH) was prepared either from packaged smoked salmon slices preserved under vacuum at 4°C (leaving the factory) (SSTHf) or from smoked salmon fillets sent by the French company sampled just after the smoking stage of CSS production (SSTHs). A 20-g portion was aseptically weighed in a sterile stomacher plastic bag equipped with a 63-μm porosity filter (Interscience, France), and 80 mL of sterile water were added to obtain a 5-fold dilution. The sample was then homogenized for 2 min using a stomacher device (Masticator IUL, Spain). The bag-filtered samples were divided into aliquots in sterile vials, and then stored at −80°C until their inoculation by fresh bacterial cultures. In order to limit qPCR inhibition, 1% of bovine serum albumin (BSA) fraction V (Sigma-Aldrich, France) and 4% of polyvinylpyrrolidone (PVP) (Sigma-Aldrich, France) were added to SSTH before DNA extraction (Kreader, 1996; Furet et al., 2009; Schrader et al., 2012).

### Viable and Dead Cell Preparation

The efficiency of the PMA and PMAxx treatments was assessed on *B. thermosphacta* DSM 20171 viable or dead cells in BHI broth or SSTH. The viable cells were obtained from a pre-culture in 10 mL of BHI broth at 25°C for 8 h, followed by a culture in 100 mL of BHI broth for 4 h, in order to obtain an 8 log CFU/mL exponential growth phase culture. The 100% viable cell culture in SSTH was produced as follows: 5 mL of the 8 log CFU/mL exponential growth phase BHI culture were centrifuged at 6,000 x g for 2 min, the supernatant was discarded, and the cells were re-suspended in 5 mL of SSTH (with BSA and PVP). Dead cells were obtained by heating 5 microtubes containing 1 ml of the 8 log CFU/mL exponential growth phase in BHI culture at 90°C for 15 min in a water bath. The suspensions were pooled and centrifuged at 6,000 × *g* for 2 min. The supernatant was discarded, and the cells were re-suspended in 5 mL of SSTH (with BSA and PVP). Cell viability was checked by plating on STAA agar.

### PMA and PMAxx Treatments

PMAxx^TM^ is a new and improved version of viability dye PMA (propidium monoazide) designed by Biotium (Biotium, Inc., Hayward, CA, United States). Like PMA, PMAxx^TM^ is a photo-reactive dye that binds covalently to DNA with high affinity after photolysis with visible light. The PCR cannot amplify this modified DNA. PMA and PMAxx^TM^ dyes are cell membrane-impermeants. PMAxx^TM^ is described to be more effective than PMA to inhibit DNA PCR amplification of dead cell with injured cell membranes, and therefore provides best discrimination between live and dead bacteria (Han et al., 2018). Commercial PMA and PMAxx solutions (20 mM) (VWR, France) were diluted in pure sterile water to obtain 1.5 mM working solutions kept at −20°C in light-tight microtubes. The samples to be treated were separated in 4 sterile microtubes containing each 580 μL of bacterial cell suspensions in BHI or inoculated SSTH. Twenty microliters of PMA working solution were added in 2 microtubes and 20 μL of PMAxx working solution were added in the remaining 2 tubes (50 μM final concentration). The microtubes were placed in the dark at room temperature for 10 min, and mixed occasionally for PMA/PMAxx to penetrate into the dead cells. To photoactivate PMA/PMAxx, the PMA-Lite^TM^ LED Photolysis Device (Biotium) was used as recommended by the PMAxx^TM^ manufacturer (Biotium). The PMA-Lite^TM^ LED Photolysis Device is a thermally stable blue LED light source (LED power: 60 W, output wavelength: 465–475 nm) that provides even illumination to the sides and bottoms of all vials. The microtubes were exposed to blue light for 20 min, to ensure complete cross-linking to the available DNA (free DNA or DNA from unviable cells). The content of the 2 microtubes treated with PMA was pooled and 1 mL of the suspension was conserved for further DNA extraction. The same operation was realized for the microtubes treated with PMAxx. For each cell suspension tested in BHI or in SSTH, one milliliter of viable or dead cell suspension was not treated with PMA or PMAxx and used as an untreated control.

PMA and PMAxx treatments were tested on SSTHs in the presence of mixes of dead and viable *B. thermosphacta* cell suspensions. Several mix conditions were tested: (i) 5.65 log CFU/g of dead cells mixed with 4.70, 5.70, or 6.70 log CFU/g of viable cells, and (ii) 7.65 log CFU/g of dead cells mixed with 4.70, 5.70, or 6.70 log CFU/g of viable cells. The samples were treated or not with PMA and PMAxx, as mentioned above. Two controls were also included: (i) 5.70 or 7.70 log CFU/g of viable cells treated or not with PMA/PMAxx, and (ii) 5.70 or 7.70 log CFU/g of dead cells treated or not with PMA/PMAxx.

### Extraction of Bacterial DNA

Genomic DNA extraction from bacterial cells cultured in BHI broth was performed using the Qiagen DNeasy Blood and Tissue kit (Qiagen, France). One milliliter of culture was centrifuged at 11,000 × *g* for 10 min at 4°C (Biofuge PimoR, Heraeus). The supernatant was removed. Bacterial DNA extraction was conducted on cell pellets according to the manufacturer’s instructions (Qiagen, France). A Qubit^®^ 2.0 fluorometer using a Qubit^®^ dsDNA BR Assay Kit (Life technologies, Thermo Fisher Scientific, France) was used to quantify the extracted DNA. Extracted DNAs were stored at −20°C until qPCR amplification.

For bacterial DNA extraction in SSTH, one milliliter of SSTH inoculated with *B. thermosphacta* cells was centrifuged at 11,000 × *g* for 10 min at 4°C. The supernatant was removed, and the pellet was stored at −20°C until use. DNA extraction was performed with the DNeasy PowerFood Microbial kit (Qiagen, France). Briefly, this kit is designed to isolate high-quality genomic DNA from microorganisms hosted in food. The microbial cell pellet from food was thawed and re-suspended in 450 μL of MBL lysis buffer preheated at 55°C for 10 min. The suspension was transferred to PowerBead tubes, containing beads designed for mechanical microbial-cell lysis, and shaken horizontally for 30 s on a Vortex-Genie 2 device (Scientific industries, United Sates), then on a FastPrep-24 G at 6 m/s for 30 s (MP Biomedicals, France), for mechanical lysis. Each sample was centrifuged at 10,000 × *g* for 1 min at room temperature. The supernatant was transferred to a microtube, and 100 μL of IRS solution were added. The microtube was incubated on crushed ice for 30 min. The next steps were performed as described in the Qiagen kit instruction manual. DNA samples were stored at −20°C until use. DNA extracts were diluted 1/10 before the qPCR amplification.

### Primer Design and Quantitative Real-Time PCR Assay

A new PCR primer set was designed to amplify a *rpoC* gene DNA fragment specific to *B. thermosphacta*, excluding other *Brochothrix* spp. (*B. campestris*) and closely related species such as *Listeria*. The *in silico* primer design was based on multiple alignment of the DNA-dependent RNA polymerase subunit (*rpoC*) gene sequences of *B. thermosphacta* and closely related species, available from the GenBank database (release 225.0). The *rpoC* sequences from *B. thermosphacta*, *B. campestris* and the most closely related bacterial species were aligned using the CLC DNA Workbench 6.5 (CLC bio, Aarhus, Denmark) and BioEdit sequence alignment software (Hall, 1999). Primer specificity was tested *in silico* using the basic local alignment search tool (BLAST) program [National Center for Biotechnology Information (NCBI)] and Primer BLAST (Ye et al., 2012) using Genbank release 225.0.

Two other previously described primer sets targeting the *rpoC* (Fougy et al., 2016) or *rpoB* (Illikoud et al., 2019) genes were also used (Table 2). AmplifX 2.0.7 software was used to evaluate the quality of the primers (Jullien, 2019). The annealing temperature was optimized using the temperature gradient test of the Bio-Rad CFX connect real-time PCR detection system (Bio-Rad, France).

**TABLE 2:** Description of the three sets of qPCR primers.

Target gene	Primer pair	Primer sequences (5′- 3′)	Amplicon size (bp)	Tm (°C)	References
*rpoC*	rpoC-126-F	ATACTGTACCAATGGTTGCTC	126	52	This study
	rpoC*-*126-R	CAACAGTGATAACATCAGTTAC			
*rpoC*	QSF03-BTH-F	GGACCAGAGGTTATCGAAACATTAACTG	151	56	Fougy et al. (2016)
	QSF03-BTH-R	TAATACCAGCAGCAGGAATTGCTT			
*rpoB*	rpoB-Fw1	GCGTGCATTAGGTTTCAGTACA	394	55	Illikoud et al. (2019)
	rpoB-Rev1	TCCAAGACCAGACTCTAATTGCT			

Quantitative real-time PCR amplification was performed using the Sso Advanced universal SYBR Green supermix (Bio-Rad, France). The reaction was performed in a final volume of 12.5 μL containing 1 μM of each primer, 6.25 μL of 2 X Sso Advanced universal SYBR Green Supermix, 2.75 μL of water (molecular biology grade) and 3 μL of DNA extract. DNA concentrations were standardized at 1 ng/μL for the specificity test. The amplification reaction was conducted using a Bio-Rad CFX connect real-time PCR detection system (Bio-Rad, France). The cycling parameters were as follows: initial denaturation step at 95°C for 3 min, 39 cycles of 95°C for 15 s, and 60°C for 30 s. A melting curve from 65 to 95°C was determined after the last amplification cycle and at a temperature transition rate of 0.5°C/3 s. Quantification cycles (Cq) values were automatically obtained by the Biorad CFX Manager software program. All amplification reactions were run in triplicate in three independent assays.

### Efficiency of the Quantitative Real-Time PCR

The efficiency of the qPCR was evaluated by using three different sample preparations. The first two were obtained from *B. thermosphacta* BHI culture: DNA was extracted from a 10-fold serial dilution range of *B. thermosphacta* BHI culture (7 to 2 log CFU/mL) (BHI culture extract), or from an 8 log CFU/mL of *B. thermosphacta* BHI suspension. This DNA extract was then serial diluted 10-fold, corresponding to 7 to 2 log CFU/mL (diluted DNA extract). The third sample preparation corresponded to DNA extracted from SSTHf inoculated with *B. thermosphacta* from 7 to 4 log CFU/mL. The *rpoC* and *rpoB* sequences were amplified using the three different species-specific primers used in this study in the qPCR conditions described above. The linear standard curves were generated by plotting the Cq values *versus* log CFU/mL to determine the analytical efficiency of the qPCR assay. The efficiency of the real-time PCR assay was calculated from the slope (m) of the standard curve according to equation E = 10^(–1/m)^−1. The qPCR was performed in three independent assays under the same conditions, using three replicates of each template concentration. The linearity of the standard curve was expressed as a coefficient of determination (r^2^).

### Limit of Detection (LOD)

The LOD was established in SSTHf for the two primer sets: rpoC-126-F/R (designed in this study) and rpoB-Fw1/Rev1. Ten replicates of 10-fold serial dilutions of *B. thermosphacta* inoculated in SSTHf from 7 to 1 log CFU/mL were prepared. DNA extracts from these inoculated samples were used as qPCR templates in triplicate. Each Cq datum corresponding to the amplification of a PCR product whose melting temperature corresponded to that of the studied gene was considered as a positive signal. The LOD was established by comparing the positive signals and the enumeration of cells on STAA agar and analyzed by a Probit model (Kralik and Ricchi, 2017) with R version 3.3.2 (2016-10-31) (The R Foundation for Statistical Computing). The *p*-value was fixed at 0.05 to detect the gene with 95% probability.

### Statistical Analysis

The Shapiro-Wilk’s test was used to check the normality of the data. Student’s *t*-test or Wilcoxon’s test were applied to determine differences between conditions tested (treated vs. untreated cultures, PMA vs. PMAxx treatments, differences of amplification between the 3 primer sets) on viable or dead *B. thermosphacta* cells in BHI broth or SSTH. Homoscedasticity was checked on the values of the standard curves obtained in SSTH using Levene’s test. ANOVA was used to control the significant statistical differences between these standard curves. ANOVA, followed by Tukey’s multiple comparison of means test was used to analyze the quantification of viable *B. thermosphacta* in cold-smoked salmon fillet sampled after the smoking step. The significant threshold of 5% was established for all the statistical tests. The tests were performed using R version 3.3.2 (2016-10-31) (The R Foundation for Statistical Computing) and the XLSTAT add-in software (version 2020) in Microsoft Excel software 2016.

## Results

### Primer Design

A new PCR primer set was designed to amplify a fragment of the *B. thermosphacta rpoC* gene encoding a DNA-dependent RNA polymerase subunit. The *in silico* primers design was based on multiple alignment of *B. thermosphacta rpoC* gene sequences and on those of closely related species available from the GenBank database. Based on this analysis, a region of the *rpoC* gene was selected as a target of the primers design to specifically distinguish *B. thermosphacta* from other related bacterial species examined in this study. The forward primer rpoC-126-F (5′-ATACTGTACCAATGGTTGCTC-3′) matched positions 3005 to 3025, and the reverse primer rpoC-126-R (5′-CAACAGTGATAACATCAGTTAC-3′) matched positions 3109 to 3130 of the *B. thermosphacta* DSM 20171 type strain *rpoC* gene (accession number X89231), to amplify a specific 126-bp fragment. Two other previously described primer sets targeting the *rpoC* or *rpoB* genes were used. Firstly, the primers QSF03-BTH-F/QSF03-BTH-R targeting the *rpoC* gene (accession number X89231), with a specific 151-bp amplified fragment (Fougy et al., 2016). The forward primer matched positions 2494 to 2521, and the reverse primer matched positions 2621 to 2644 of the *B. thermosphacta* DSM 20171 type strain. Secondly, the primers rpoB-Fw1/Rev1 targeting a specific region of *B. thermosphacta rpoB* gene were used (Illikoud et al., 2019). The forward primer rpoB-Fw1 matched positions 609 to 630, and the reverse primer rpoB-Rev1 matched positions 980 to 1022 of the *B. thermosphacta* DSM 20171 type strain *rpoB* gene (Gene ID 29820965), with a specific 394-bp amplified fragment. We selected this later *rpoB* primer, amplifying a longer fragment, because the PCR inhibition effect by PMA can be dependent on the length of the amplification product (Martin et al., 2013).

*In silico* studies with BLAST program and Primer BLAST using Genbank release 225.0. demonstrated that the primers developed in the present study (rpoC-126-F/R) and those developed by Fougy et al. (2016) and Illikoud et al. (2019) were *B. thermosphacta*-specific. Furthermore, the quality assessment of the designed primers using AmplifX 2.0.7. software revealed no hairpin loop, dimer or duplex formation. The hybridization temperature of the primers was optimized using the temperature gradient test of the Bio-Rad CFX connect real-time PCR detection system (Bio-Rad, France). The 3 sets of primers were tested for qPCR specificity and efficiency.

### Specificity of the qPCR Assay

The specificity of the qPCR assay was assessed by inclusivity and exclusivity tests using 26 different bacterial strains including six *B. thermosphacta* strains and 20 non-targeted strains frequently associated with *B. thermosphacta* in seafood products. DNAs were extracted from pure cultures in the appropriate growth conditions (Table 1). The specificity of the three primer sets was verified using the qPCR Cq values obtained with 3 ng of DNA template (Table 3). All the Cq values of the *B. thermosphacta* strains were lower than 16.76 ± 0.05 (rpoC QSF03-BTH-F/R, strain CD340). No amplification signal was observed for six non-targeted strains when the rpoB-Fw1/Rev1 primers were used. The lowest Cq value for a non-targeted strain was 29.48 ± 0.18 (*Staphylococcus epidermidis* CIP 68.21 with the rpoC-126-F/R primers); it was 12.72 Cq above the highest value of the targeted strains, and was considered as the unspecific detection threshold. The primers rpoC QSF03-BTH-F/R produced a single melting peak with Tm values of 81 ± 0.50°C while the rpoB-Fw1/Rev1 and the rpoC-126-F/R primers generated a single melting peak with a Tm value of 82 ± 0.50°C. This confirmed the high specificity levels of the three sets of primers. The three primer sets correctly identified all *B. thermosphacta* strains, including reference and food-isolate strains. As none of the tested sets gave specific amplification (<29.48 Cq) when DNA from non-targeted strains was used, the three sets were considered as species-specific by q-PCR.

**TABLE 3:** Primer specificity (Cq values).

Strains	rpoC-126-F/R		QSF03-BTH-F/R		rpoB-Fw1/Rev1	
	Cq values^a^	Range of Tm	Cq values^a^	Range of Tm	Cq values^a^	Range of Tm
*Brochothrix thermosphacta* DSM 20171^T^	15.32 ± 0.10	82–82.5	15.45 ± 0.06	81	14.67 ± 0.07	82
*Brochothrix thermosphacta* 19/R/663	14.42 ± 0.33	82–82.5	14.63 ± 0.05	81	13.94 ± 0.07	82
*Brochothrix thermosphacta* CD 340	16.43 ± 0.09	82–82.5	16.76 ± 0.05	81–81.5	16.43 ± 0.16	82–82.5
*Brochothrix thermosphacta* EBP 3084	15.26 ± 0.22	82–82.5	15.44 ± 0.04	81	14.60 ± 0.18	82
*Brochothrix thermosphacta* TAP 125	15.28 ± 0.18	82–82.5	15.44 ± 0.08	81	14.62 ± 0.14	82
*Brochothrix thermosphacta* EBP 3017	14.82 ± 0.21	82–82.5	14.93 ± 0.06	81	14.00 ± 0.22	82
*Brochothrix campestris* 4712^T^	30.49 ± 0.23	81.5	31.03 ± 0.17	80.5–81	31.52 ± 0.30	82
*Carnobacterium maltaromaticum* 19/R/671	34.60 ± 0.48	79.5–81.5	36.04 ± 1.10	80.5–82	35.54*	NA
*Carnobacterium divergens* NCDO 2763^T^	35.79 ± 0.47	78.5–82.5	35.43 ± 0.65	80–83.5	NA	NA
*Enterococcus faecalis* ATCC 19433^T^	35.15 ± 0.68	79–81	35.02 ± 1.14	80.5–81	36.14 ± 1.39	81.5
*Enterococcus faecium* CIP 54.33	34.57 ± 0.79	80–81	34.37 ± 0.67	79.5–80.5	34.74 ± 1.35	80.5–81
*Escherichia coli* CIP 53.125	31.39 ± 0.60	87.5	36.88 ± 0.62	75.5–81	NA	NA
*Companilactobacillus alimentarius* DSM 20181	34.77 ± 1.09	73.5–81.5	36.14 ± 0.74	80.5–81	36.02 ± 0.76	82
*Latilactobacillus sakei subsp. sakei DSM 20017^T^*	35.59 ± 0.63	79.5–81.5	36.12 ± 1.19	81–83.5	NA	NA
*Lactococcus piscium* 19/R/684	34.56 ± 0.92	79.5–82	35.46 ± 1.44	80.5–81.5	35.05 ± 0.84	82
*Lactococcus raffinolactis* Oniris MIP 2453	36.22 ± 0.10	79.5–80	NA	NA	NA	NA
*Listeria monocytogenes* DSM 12464^T^	35.61 ± 0.66	79.5–83	34.12 ± 1.04	84–84.5	36.51 ± 0.99	82
*Listeria monocytogenes* CIP 78.35	35.37 ± 0.49	79.5–81.5	33.39 ± 1.20	84	35.53*	NA
*Listeria grayi* CIP 68.18^T^	365.09 ± 0.22	81.5	35.01 ± 1.26	80.5–81	36.50 ± 0.46	82
*Listeria innocua* CIP 80.11^T^	35.71 ± 0.62	79.5–83	36.32*	81	37.07*	76
*Photobacterium phosphoreum* CIP 102511^T^	34.59 ± 1.05	81.5–83	35.31 ± 1.48	80.5–81	36.47 ± 0.79	82
*Pseudomonas fluorescens* CIP 69.13^T^	34.35 ± 0.50	91	NA	NA	NA	NA
*Psychrobacter sp.* 19/R/675	35.09 ± 0.40	80.5–82.5	NA	NA	NA	NA
*Serratia liquefaciens* ATCC 27592^T^	34.91 ± 0.30	82.5	NA	NA	NA	NA
*Shewanella baltica* 19/R/670	32.55 ± 0.38	82–87	NA	NA	NA	NA
*Staphylococcus epidermidis* CIP 68.21	29.48 ± 0.18	81.5–82	29.74 ± 0.22	80.5–81	29.98 ± 0.25	82
*NTC*	34.87 ± 1.99	72–83	38.58*	76.50	NA	NA

^a^Mean and standard deviation of three Cq values (obtained with 3 ng of DNA template). NTC, no template control; NA, no amplification. *no standard deviation calculated (only one Cq available).

### qPCR Efficiency Using DNA Extracted From BHI Cultures

The qPCR efficiency of the three primer sets tested on BHI culture extracts and on diluted DNA extracts was calculated from the standard curves plotting the mean Cq values (three replicates) *versus* log CFU/mL (Table 4). The linear coefficient of determination (r^2^) ranged from 0.990 (BHI culture extracts; rpoB-Fw1/Rev1) to 1 (diluted DNA extracts; rpoC-126-F/R). Using the primers rpoC-126-F/R (designed for this study), the efficiency levels were 97.45 and 92.58% for diluted DNA extracts and BHI culture extracts, respectively. Using the two other primer set, efficiency was lower and ranged from 89.46 to 92.80%.

**TABLE 4:** qPCR efficiency.

	rpoC-126-F/R			QSF03-BTH-F/R			rpoB-Fw1/Rev1		
	Equation^a^ y =	Efficiency (%)	r^2^	Equation^a^ y =	Efficiency (%)	r^2^	Equation^a^ y =	Efficiency (%)	r^2^
BHI culture extracts	−3.5136x + 40.558	92.58	0.997	−3.5898x + 41.019	89.92	0.996	−3.5419x + 42.158	91.57	0.990
Diluted DNA extracts	−3.3845 + 40.947	97.45	1	−3.5074x + 41.542	92.80	0.998	−3.6035x + 41.882	89.46	0.995
Smoked salmon tissue homogenate	−3.2408x + 44.396	103.50	0.998	Not determined			−3.5789 + 46.805	90.29	0.998

*^a^equations derived from 3 replicates.*

### qPCR Efficiency and Limit of Detection Using DNA Extracted From Smoked Salmon Tissue Homogenates

The rpoC-126-F/R and rpoB-Fw1/Rev1 primer sets, showing the highest qPCR efficiency on BHI culture extracts, were chosen to evaluate efficiency on DNA extracted from SSTHf. DNA was extracted from SSTHf inoculated with *B. thermosphacta* DSM 20171 from 7 to 4 log CFU/mL (SSTHf extract). The efficiency of the qPCR amplification using rpoC-126-F/R and rpoB-Fw1/Rev1 (Table 4) was calculated from standard curves (data not shown) generated by plotting the mean Cq values from three replicates vs. log CFU/mL. When the rpoC-126-F/R primers (designed in this study) were used, an efficiency of 103.50% was obtained. Lower efficiency (90.29%) was calculated with the rpoB-Fw1/Rev1 primers. The linear coefficient of determination (r^2^) was 0.998 with both primer sets.

The limit of detection (LOD) was calculated using a Probit model (Kralik and Ricchi, 2017), only based on SSTHf. It was established at 3.34 log CFU/mL (2.20 × 10^3^ CFU/mL) for the rpoC-126-F/R primers, corresponding to a load of 4.04 log CFU/g of salmon (1.10 × 10^4^ CFU/g), with a 95% probability to detect the gene. For the rpoB-Fw1/Rev1 primers, the LOD was 3.15 log CFU/mL (1.41 × 10^3^ CFU/mL), corresponding to a load of 3.85 log CFU/g of salmon (7.06 × 10^3^ CFU/g).

### PMA-qPCR Quantification of Viable *B. thermosphacta* in BHI Culture

To test the efficiency of the PMA and PMAxx treatments, viable or dead pure-culture *B. thermosphacta* cells were 10-fold serially diluted in BHI (7 to 3 log CFU/mL), and their DNA was extracted after PMA or PMAxx treatment. The qPCR assays using the three primer sets were used to quantify viable *B. thermosphacta*. The results were expressed in delta-Cq (i.e., differences in Cq values between PMA/PMAxx-treated cells/untreated dead cells on the one hand, and Cq values of untreated 100% viable cells on the other hand).

When viable cells were treated with PMA or PMAxx, the delta Cq values were low with the three primer sets (Supplementary Data 1). The minimum and maximum delta-Cq values were obtained with rpoB-Fw1/Rev1 (0.01 ± 0.46 vs. 1.34 ± 0.62 for 4 log CFU/mL of viable PMA-treated cells and 5 log CFU/mL of viable PMAxx-treated cells, respectively). These low delta-Cq values indicated no significant difference in the detection and quantification of *B. thermosphacta* between viable untreated and dye-treated cells. These observations were statistically in accordance with Wilcoxon’s test (*p* > 0.4). Moreover, there was no significant difference between the delta-Cq values following treatment with the two PMA and PMAxx dyes (*p* > 0.16) or between the three primer sets (*p* > 0.18).

When dead cells were used, delta Cq values were higher with PMA/PMAxx-treated cells than with untreated cells. The minimum delta-Cq values were obtained for the 6 log CFU/mL of dead PMA-treated cells with rpoC-126-F/R and rpoC QSF03-BTH-F/R (5.92 ± 0.82 and 5.92 ± 0.83, respectively). The maximum delta-Cq value (8.57 ± 0.61) was obtained with rpoC QSF03-BTH-F/R for the 7 log CFU/mL of dead PMAxx-treated cells. Interestingly, we failed to calculate delta-Cq values for any cell concentration range when rpoB-Fw1/Rev1 was used with PMA/PMAxx-treated cells, indicating that the Cq values were either beyond the unspecific detection threshold (i.e., 29.48) or even none amplification signal was detected. Thus, there was no significant difference between the two *rpoC* primer sets, while there was a significant difference between the *rpoC* primer sets and the *rpoB* primer set (*p* < 0.005). Finally, there was no significant difference in delta-Cq values between the two PMA/PMAxx dyes (*p* > 0.16), similarly to viable cells.

Because the delta-Cq values obtained with the 2 sets of *rpoC* primers were similar and the efficiency of the rpoC-QSF03-BTH-F/R primers was lower, we decided to choose rpoC-126-F/R and rpoB-Fw1/Rev1 for further *B. thermosphacta* PMA-qPCR quantification in SSTH.

### PMA-qPCR Quantification of Viable *B. thermosphacta* in Smoked Salmon Tissue Homogenates (SSTHf)

We used the rpoC-126-F/R and rpoB-Fw1/Rev1 primer sets to quantify viable *B. thermosphacta* in SSTHf by qPCR. Suspensions of *B. thermosphacta* viable or dead cells in SSTHf were 10-fold serially diluted in SSTHf (7 to 4 log CFU/mL), and their DNA was extracted after PMA or PMAxx treatment.

When viable cells were treated with PMA or PMAxx, delta Cq values were low with the two primer sets (Supplementary Data 1), and the highest value was obtained for 5 log CFU/mL of viable PMA-treated cells with the rpoC-126-F/R primer set (1.20 ± 0.41). The delta-Cq values of viable PMA/PMAxx-treated cells were not significantly different from those of untreated viable cells (*p* > 0.05). Moreover, as previously shown in BHI broth, there was no significant difference in delta-Cq values between the two PMA/PMAxx dyes (*p* > 0.28) or between the two primer sets (*p* > 0.36). Interestingly, when dead cells were tested, none of the primer sets allowed determining delta-Cq on PMA- or PMAxx- treated cells because Cq values were beyond the detection limit. Therefore, no quantification was possible after dye treatment. When dead cells were not treated with the dyes, the difference between the Cq values of untreated dead cells *vs*. untreated viable cells was not significantly different (*p* > 0.1), even if the delta Cq ranged from 1.68 ± 0.77 to 2.66 ± 1.32.

### Quantification of Viable *B. thermosphacta* in Cold-Smoked Salmon Fillet Sampled After the Smoking Step (SSTHs)

**FIGURE 1:**
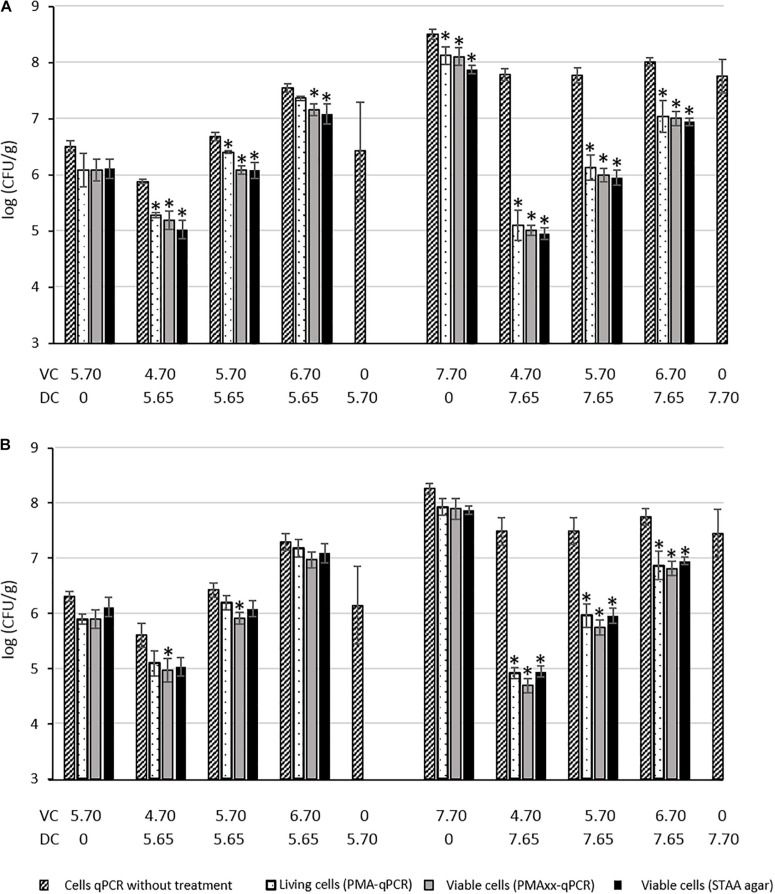
Quantification of dead (DC) and viable (VC) *B. thermosphacta* cells mixed in smoked salmon tissue homogenate (SSTHs) with plating enumeration on STAA medium, and PMA or PMAxx qPCR using the rpoC-126F/R **(A)** or rpoB-Fw1/Rev1 **(B)** primer sets. Results expressed in log CFU/g. *significant difference between quantification results of untreated cells obtained by qPCR method and treated cells quantified by qPCR method or plating method.

The PMA and PMAxx treatments were tested on SSTHs in the presence of mixes of dead and viable *B. thermosphacta* cell suspensions. The ability of PMA/PMAxx-qPCR to discriminate viable cells from dead cells in the mixes was evaluated by establishing the relationship between Cq values obtained by qPCR (after PMA/PMAxx treatments or without treatment) and bacterial counts of untreated cells on STAA agar. The results are presented in Figures 1A,B and in Supplementary Data 2. ANOVA and the Tukey’s multiple comparison of means were used to analyze the results. The significance level was set for *p*-values <0.05.

For both primer sets, the quantification of 5.70 log CFU/g of PMA/PMAxx-treated viable cells was consistent with the quantification of untreated viable cells, with a non-significant difference (*p* > 0.05). Likewise, non-significant difference was observed between the quantification of 7.70 log CFU/g of PMA/PMAxx-treated viable cells and the untreated viable cells with rpoB-Fw1/Rev1 but a significant difference was observed with the rpoC-126-F/R primers. As previously shown in SSTHf on 5.70 or 7.70 log CFU/g of dead cells, quantification was only possible with untreated cells, whereas no quantification was possible once the cells were treated with PMA or PMAxx, suggesting that PMA and PMAxx inhibit dead cell DNA amplification.

In the mixes containing 5.65 log CFU/g of dead cells and 4.70 or 5.70 log CFU/g of viable cells, no significant difference was highlighted between PMA-treated and untreated cells using rpoB-Fw1/Rev1 but a significant difference was highlighted between PMAxx-treated and untreated cells. When rpoC-126-F/R was used, a significant difference was observed between PMA/PMAxx-treated and untreated cells in those mixes. No treatment-related difference was noticed in the mixes containing 5.65 log CFU/g of dead cells and 6.70 log CFU/g of viable cells with rpoB-Fw1/Rev1 whereas a significant difference was observed between PMAxx-treated and untreated cells with rpoC-126-F/R. In the mixes containing 7.65 log CFU/g of dead cells and 4.70, 5.70, or 6.70 log CFU/g of viable cells, the efficiency of PMA/PMAxx was more obvious: differences between treated and untreated cells were significant with both primer sets, and viable cells were preferentially amplified and quantified. This shows again the good ability of PMA and PMAxx to inhibit DNA amplification from dead cells.

## Discussion

The detection and quantification of unviable cells is admittedly a question of molecular methods targeting the cell DNA directly without culturing steps. This requires methods that discriminate viable cells from unviable cells in biological samples such as food products (Kumar and Ghosh, 2019). In the field of food spoilage, *B. thermosphacta* is considered as one of the main bacteria, able to spoil many meat and seafood products whatever the packaging (i.e., under air, vacuum, or modified atmosphere) (Illikoud et al., 2018a). *B. thermosphacta* is even considered as ubiquitous along the food chain because it was also isolated from food factory surfaces (Nychas et al., 2008). Thus, it can be subjected to the environmental and food processing stresses likely to lead to unviable state, making it difficult to detect and quantify it from food products.

We developed an assay aimed at quantifying viable *B. thermosphacta* using viability dyes (PMA or PMAxx) and the targeting of a single-copy gene (*rpoC* or *rpoB*). After designing the primers *in silico*, we determined their specificity and compared it with that of two other single-copy gene primer sets from previous studies specifically designed to identify *B. thermosphacta* (Fougy et al., 2016; Illikoud et al., 2019). Using the real-time qPCR inclusivity and exclusivity tests, including DNA extracted and purified from 6 *B. thermosphacta* strains and 20 non-targeted closely related strains such as *B. campestris*, we demonstrated the specificity of the three primer sets, without misdetection or misquantification. The difference between targeted and untargeted strains was at least 12.72 Cq. The efficiency of the qPCR assays with the three primer sets was then assessed from the standard curves [Cq = f(CFU (log/mL))]. The most efficient primer set to quantify *B. thermosphacta* from diluted DNA extracts and BHI culture extracts was rpoC-126-F/R, with satisfactory efficiency levels of 97.45% (r^2^ = 1) and 92.58% (r^2^ = 0.997), respectively.

We further investigated the efficiency of PMA and PMAxx treatments to quantify viable *B. thermosphacta* cells in BHI medium. As described in many previous studies (Zeng et al., 2016), our results showed that a viable dye treatment was clearly relevant to discriminate viable from unviable bacterial cells. The delta-Cq for dead *B. thermosphacta* cells was higher when PMA or PMAxx treatments were applied than it was for untreated cells, with a difference of at least 3.48 in the delta-Cq values between PMA-treated cells and untreated cells at 6 log CFU/mL when rpoC QSF03-BTH-F/R was used. These results confirm that the number of amplification cycles is higher after dye treatment when it comes to detecting and quantifying dead *B. thermosphacta* cells rather than viable cells because the amount of amplifiable target DNA is very low when dead cells are treated with dyes. The most important effect of the two dye treatments was also obvious with the *rpoB* primer set: no delta-Cq values were calculable from dead treated cells, meaning that no amplification was possible in these conditions. In the absence of dye treatment, amplification was still detectable up to 4 log CFU/mL, with a low associated delta-Cq value (1.76 ± 0.70).

Regarding the two *rpoC* primer sets, they gave quite similar results on viable and dead cells. However, whatever the primer set, there was no significant difference between the PMA and PMAxx treatments, with similar delta-Cq values for the two conditions. Because the delta-Cq values obtained with both *rpoC* primer sets were quite similar and rpoC-126F/R was slightly more efficient than rpoC-QSF03-BTH-F/R was, we only kept rpoC-126F/R for the further tests on SSTH.

The good efficiency of rpoC-126F/R was confirmed on smoked salmon tissue homogenate (SSTHf), with 103.50% (r^2^ = 0.998), while rpoB-Fw1/Rev1 showed 90.29% (r^2^ = 0.998) efficiency. We also determined the limit of detection (LOD) of the qPCR assays on SSTHf, but not on BHI, to be closest to a real food product. The LODs were established at 4.04 and 3.85 log CFU/g of smoked salmon for rpoC-126F/R and rpoB-Fw1/Rev1, respectively. We considered these LODs as relevant according to the food spoilage behavior of *B. thermosphacta*. In most of the reported cases of food spoilage, the amount of *B. thermosphacta* in the spoiled foods was above 7 log CFU/g when spoilage was perceived by sensory analysis panelists (Illikoud et al., 2018a). With our *rpoC* or *rpoB* qPCR assays, we detected the presence of *B. thermosphacta* around 3 log before spoilage perception. This could give smoked salmon producers the opportunity to redirect their matrices before spoilage by *B. thermosphacta* to other transformation processes with a strong stabilization step – e.g., pasteurization – to kill the spoilage agent and limit food losses. The results of the qPCR assays after PMA or PMAxx treatment of viable smoked salmon tissue homogenate were similar to those of viable cells cultured in BHI, with low delta-Cq values and no significant difference between the primer sets. Nevertheless, the effect of the dye treatments on dead cells was more obvious than it was on BHI-cultured cells because no delta-Cq value was calculable on treated dead cells for either primer set. Therefore, no amplification was possible with these primer sets after dye treatment of dead cells. By contrast, amplification was still detectable down to 4 log CFU/mL in untreated cells, with a low associated delta-Cq value (1.74 ± 0.52 and 2.66 ± 1.32 for the *rpoC* and *rpoB* primer sets, respectively).

The efficiency of PMA/PMAxx-qPCR to discriminate viable *B. thermosphacta* cells from dead cells was also confirmed on industrially processed cold-smoked salmon fillets after the smoking step (SSTHs). The DNA amplification of 5.70 or 7.70 log CFU/g of *B. thermosphacta* dead cells inoculated on SSTHs was inhibited by PMA and PMAxx, whereas qPCR amplification was consistently detectable in the absence of dye treatment. This efficiency was even more obvious in mixed viable/dead-cell conditions, with significantly different quantifications between the treated and the untreated cells with both primer sets. However, we did not find any significant difference between the two primer sets, except for the mixes containing 5.65 log CFU/g of dead cells + 4.70 or 5.70 log CFU/g of PMA/PMAxx-treated viable cells and 5.65 log CFU/g of dead cells + 6.70 log CFU/g of PMAxx-treated viable cells where rpoC-126F/R better discriminated between treated and untreated cells. Moreover, we did not find a better PCR inhibition by PMA or PMAxx with the longer *rpoB* amplicon (394 bp), as previously described by Martin et al. (2013), compared with the shorter *rpoC* amplicons (126 or 151 bp). Consequently, our results suggest using rpoC-126-F/R coupled with PMA or PMAxx treatment to specifically quantify *B. thermosphacta* with good efficiency and reliable discrimination between viable and dead cells in cold-smoked salmon samples.

In the last two decades, many studies have described the interest of molecular methods such as real-time quantitative PCR to detect and quantify a lot of bacterial species in food or other ecosystems instead of the classical culture methods (Postollec et al., 2011; Franco-Duarte et al., 2019), but also the usefulness of viability dyes such as EMA or PMA to discriminate viable cells from unviable or dead cells (Elizaquível et al., 2014; Zeng et al., 2016). Regarding *B. thermosphacta*, only one study demonstrates the interest of PMA coupled with 16S rDNA-qPCR to detect and quantify this spoilage bacterium in cooked peeled shrimp (Mamlouk et al., 2012). These authors found a better LOD than ours (2.07 log CFU/g of cooked shrimp) with a similar efficiency of 105.3% (r^2^ = 0.999). Nevertheless, their LOD was not determined from the food matrix, as it is in the present study, but from purified genomic *B. thermosphacta* DNA serially diluted 10-fold in sterile saline solution and subjected to real-time PCR amplification. Consequently, these conditions reduce the potential presence of PCR inhibitors likely present in food matrices and able to influence on qPCR efficiency, hence a shift of the LOD (Sidstedt et al., 2020).

The new viability dye PMAxx was developed recently. It improves the discrimination between viable and unviable cells (Randazzo et al., 2016). Few studies have been conducted with this new dye so far (Truchado et al., 2020) used it to detect and quantify VBNC *L. monocytogenes* cells in wash water from processed fruit and vegetables. It has also been used to quantify *L. monocytogenes* in chocolate liquor, corn flakes, and dry-roasted, shelled pistachios (Ly et al., 2020), quantify viable and non-viable *Salmonella* from a poultry environment (Zhang et al., 2020), quantify viable *Campylobacter* in raw milk (Wulsten et al., 2020), detect *Clavibacter michiganensis* in a viable but non-culturable state in tomato (Han et al., 2018), discriminate between viable and membrane-damaged cells of the plant pathogen *Xylella fastidiosa* (Sicard et al., 2019) or quantify VBNC *Vibrio parahaemolyticus* in raw shrimp (Cao et al., 2019). Among all these studies, three of them have compared this new dye with other available dyes, notably EMA or PMA. Two of them have demonstrated a higher suitability of PMAxx than PMA to quantify bacteria in food matrices (Han et al., 2018; Cao et al., 2019). By contrast, another study found PMA more suitable than EMA and PMAxx to detect and quantify viable bacterial cells (Wulsten et al., 2020). Other authors even reported the use of a combination of EMA and PMAxx to reduce the DNA signal from dead cells (with intact and damaged membranes) and viable cells with inactive membranes (Truchado et al., 2020). The use of the EMA-PMAxx combination yielded a more accurate estimation of viable *L. monocytogenes* cells. Our results did not allow us to clearly distinguish between PMAxx and PMA in their efficiency to discriminate viable *B. thermosphacta* cells from unviable ones in cold-smoked salmon: they gave similar results in most of the tested conditions.

In conclusion, we developed a rapid, specific and efficient *rpoC* PMA/PMAxx-qPCR method to detect and quantify *B. thermosphacta* in pure culture and in cold-smoked salmon samples, with efficient discrimination between viable and dead cells. This qPCR method specifically and quickly enumerates *B. thermosphacta* (within 3–4 h compared with 48 h for the STAA culture method). Moreover, this PMA/PMAxx-qPCR could be a relevant tool for smoked salmon producers to (i) quickly detect and quantify *B. thermosphacta* in their products early enough before spoilage is perceived and (ii) limit food losses. Finally, this *rpoC* PMA/PMAxx-qPCR method could be used to detect and quantify *B. thermosphacta* in other seafood products and even in other food matrices such as meat products.

## Data Availability Statement

The original contributions presented in the study are included in the article/Supplementary Material, further inquiries can be directed to the corresponding author/s.

## Author Contributions

AB-A, EJ, HP, and XD conceived and designed the experiments. AB-A contributed to reagents, materials, and analysis tools. AB-A and SS performed the experiments. AB-A, EJ, and HP wrote the manuscript. All authors analyzed the data, contributed to the article, and approved the submitted version.

## Conflict of Interest

The authors declare that the research was conducted in the absence of any commercial or financial relationships that could be construed as a potential conflict of interest.
