# Supplementary material for: Quantification of Viable Brochothrix thermosphacta in Cold-Smoked Salmon Using PMA/PMAxx-qPCR
Source: Front Microbiol. 2021 Jul 14;12:654178. doi: 10.3389/fmicb.2021.654178 (PMC8316974; doi:10.3389/fmicb.2021.654178)
Supplement: Supplementary file 1 [file Table_1.DOCX]

**Supplementary data S1:**

**Delta Cq of viable and dead cells of Brochothrix thermosphacta in BHI broth and in smoked-salmon tissue homogenate.**

|  | **Cells** | **BHI** | | | | | **Smoked-salmon tissue homogenate** | | | | |
| --- | --- | --- | --- | --- | --- | --- | --- | --- | --- | --- | --- |
| **Primer sets** | **log (CFU/ml)** | **Viable cells** | | **Dead cells** | | | **Viable cells** | | **Dead cells** | | |
|  |  | **PMA** | **PMAxx** | **PMA** | **PMAxx** | **Untreated** | **PMA** | **PMAxx** | **PMA** | **PMAxx** | **Untreated** |
| rpoC-126F/R | **7** | 0.74±1.05 | 0.44±0.33 | 6.85±0.99 | 8.01±0.51 | 1.68±0.46 | 0.91±0.40 | 0.67±0.55 | No ΔCq | No ΔCq | 2.42±0.69 |
|  | **6** | 0.21±0.37 | 0.58±0.27 | 5.92±0.82 | 7.08±0.28 | 2.38±0.59 | 1.14±0.65 | 0.80±0.37 | No ΔCq | No ΔCq | 1.94±0.13 |
|  | **5** | 0.47±0.33 | 1.00±0.48 | 6.60±1.15 | 6.77±0.64 | 2.12±0.34 | 1.20±0.41 | 0.69±0.19 | No ΔCq | No ΔCq | 1.68±0.77 |
|  | **4** | 0.14±0.49 | 0.86±0.77 | No ΔCq | No ΔCq | 1.40±0.41 | 0.96±0.56 | 0.61±0.40 | No ΔCq | No ΔCq | 1.74±0.52 |
|  | **3** | -0.74±0.81 | 0.58±0.56 | No ΔCq | No ΔCq | No ΔCq |  |  |  |  |  |
| QSF03-BTH-F/R | **7** | 0.77±1.00 | 0.43±0.41 | 7.27±0.98 | 8.57±0.61 | 1.70±0.45 |  |  |  |  |  |
|  | **6** | 0.53±0.28 | 0.76±0.48 | 5.92±0.83 | 7.35±0.29 | 2.44±0.58 |  |  |  |  |  |
|  | **5** | 0.58±0.42 | 1.20±0.65 | 7.00±1.48 | 7.38±0.83 | 2.23±0.33 |  |  |  |  |  |
|  | **4** | -0.09±0.19 | 0.50±0.43 | No ΔCq | No ΔCq | 1.40±0.57 |  |  |  |  |  |
|  | **3** | -0.58±0.99 | 0.82±0.41 | No ΔCq | No ΔCq | No ΔCq |  |  |  |  |  |
| rpoB-Fw1/Rev1 | **7** | 0.54±0.50 | 0.88±0.63 | No ΔCq | No ΔCq | 2.00±1.03 | 0.89±0.25 | 0.54±0.12 | No ΔCq | No ΔCq | 2,58±0.45 |
|  | **6** | 0.38±0.35 | 0.95±0.19 | No ΔCq | No ΔCq | 3.30±1.34 | 1.18±0.69 | 0.74±0.45 | No ΔCq | No ΔCq | 2,32±0.25 |
|  | **5** | 0.65±0.34 | 1.34±0.62 | No ΔCq | No ΔCq | 2.70±0.77 | 1.16±0.31 | 0.57±0.35 | No ΔCq | No ΔCq | 2,37±0.38 |
|  | **4** | 0.01±0.46 | 0.78±0.73 | No ΔCq | No ΔCq | 1.76±0.70 | 0.56±0.55 | 0.32±0.57 | No ΔCq | No ΔCq | 2,66±1.32 |
|  | **3** | No ΔCq | No ΔCq | No ΔCq | No ΔCq | No ΔCq |  |  |  |  |  |

The values are Delta Cq ± Standard Deviation. Delta Cq = Cq of samples treated with PMA, PMAxx or untreated 0% viable cells - Cq of untreated 100% viable cells. No ΔCq: Cq of samples treated with PMA, PMAxx or untreated 0% viable cells out of quantification limit.
