# Supplementary material for: Quantification of Viable Brochothrix thermosphacta in Cold-Smoked Salmon Using PMA/PMAxx-qPCR
Source: Front Microbiol. 2021 Jul 14;12:654178. doi: 10.3389/fmicb.2021.654178 (PMC8316974; doi:10.3389/fmicb.2021.654178)
Supplement: Supplementary file 2 [file Table_2.DOCX]

**Supplementary data S2:**

**Quantification of viable and dead *B. thermosphacta* cells mixed in smoked-salmon tissue homogenates with plating enumeration on STAA medium and PMA or PMAxx q-PCR using rpoC-126F/R or rpoB-Fw1/Rev1 primer sets. Results expressed in log CFU/g.**

|  | Cells |  | qPCR rpoC-126F/R | | | qPCR rpoB-Fw1/Rev1 | | | | Plating |
| --- | --- | --- | --- | --- | --- | --- | --- | --- | --- | --- |
| Dead | **Viable** | **Total** | **No treatment** | **PMA** | **PMAxx** | **No treatment** | **PMA** | | **PMAxx** |  |
|  |  |  |  |  |  |  | |  |  |  |
|  | **5.70** | **5.70** | 6.51±0.10 | 6.08±0.30 | 6.09±0.19 | 6.30±0.09 | | 5.90±0.09 | 5.90±0.17 | 6.11±0.17 |
| 5.65 | **4.70** | **5.70** | 5.88±0.03 | 5.28±0.05^a^ | 5.19±0.16^a^ | 5.61±0.22 | | 5.10±0.23 | 4.97±0.21^b^ | 5.03±0.17^a^ |
|  | **5.70** | **5.98** | 6.68±0.07 | 6.41±0.02^a^ | 6.09±0.08^a^ | 6.42±0.13 | | 6.19±0.13 | 5.91±0.10^b^ | 6.08±0.14^a^ |
|  | **6.70** | **6.74** | 7.55±0.07 | 7.36±0.04 | 7.16±0.11^a^ | 7.29±0.15 | | 7.19±0.16 | 6.97±0.14 | 7.09±0.18^a^ |
| 5.70 |  | **5.70** | 6.43±0.86 |  |  | 6.15±0.70 | |  |  |  |
|  | **7.70** | **7.70** | 8.51±0.09 | 8.12±0.16^a^ | 8.11±0.15^a^ | 8.26±0.10 | | 7.93±0.15 | 7.90±0.19 | 7.88±0.08^a^ |
| 7.65 | **4.70** | **7.65** | 7.79±0.10 | 5.10±0.26^a^ | 5.01±0.09^a^ | 7.49±0.25 | | 4.92±0.10^b^ | 4.69±0.14^b^ | 4.95±0.10^a,b^ |
|  | **5.70** | **7.66** | 7.78±0.13 | 6.13±0.23^a^ | 5.99±0.12^a^ | 7.49±0.24 | | 5.96±0.22^b^ | 5.75±0.14^b^ | 5.96±0.14^a,b^ |
|  | **6.70** | **7.70** | 8.01±0.07 | 7.04±0.28^a^ | 7.00±0.13^a^ | 7.75±0.16 | | 6.87±0.26^b^ | 6.82±0.12^b^ | 6.95±0.07^a,b^ |
| 7.70 |  | **7.70** | 7.76±0.29 |  |  | 7.45±0.44 | |  |  |  |

^a^ Significant difference between results of untreated cells obtained by qPCR method and treated cells quantified by qPCR method or plating method, with rpoC-126-F/R primer set.

^b^ Significant difference between results of untreated cells obtained by qPCR method and treated cells quantified by qPCR method or plating method, with rpoB-Fw1/Rev1 primer set.
